# Development and Validation of an Extra Spindle Pole Bodies–like 1–Based Diagnostic and Prognostic Model for Hepatitis B Virus–Related Hepatocellular Carcinoma: Retrospective Cohort Study

**DOI:** 10.2196/78354

**Published:** 2025-10-22

**Authors:** Lu-Huai Feng, Hengkai Liang, Bobin Hu, Lu Wei, Qingmei Li, Tumei Su, Qianbing Yin, Yanfei Feng, Long Huang, Hongqian Liang, Minghua Su, Jianning Jiang

**Affiliations:** 1 Department of Infectious Diseases, The First Affiliated Hospital of Guangxi Medical University, 6 Shuangyong Road, Nanning, 530021, China, 86 0771-5719573; 2 Key Laboratory of Early Prevention and Treatment of Regional High Incidence Tumors (Guangxi Medical University), Ministry of Education, Nanning, China; 3 Guangxi Key Laboratory of Early Prevention and Treatment for Regional High Frequency Tumor, Nanning, China

**Keywords:** hepatocellular carcinoma, alpha-fetoprotein, ESPL1, machine learning, web calculator

## Abstract

**Background:**

Early diagnosis of hepatocellular carcinoma (HCC) in patients with chronic hepatitis B virus (HBV) is challenging. Models that combine novel biomarkers with clinical features may improve both early diagnosis and risk stratification, but few have been systematically validated.

**Objective:**

This study aimed to develop and validate an extra spindle pole bodies–like 1 (ESPL1)–based model for diagnostic discrimination of HBV-related HCC and longitudinal risk stratification in patients with chronic HBV infection.

**Methods:**

Patients with chronic HBV were consecutively recruited from the First Affiliated Hospital of Guangxi Medical University (a single-center, tertiary hospital) between January 2012 and November 2023. Patients were divided into a training set and an independent hold-out testing set. A LASSO logistic regression model was constructed to identify independent predictors and then used to develop a risk score discriminating patients with HBV-related HCC from those with chronic hepatitis B or cirrhosis. Model performance was evaluated using discrimination (C-index), calibration, and decision curve analysis. Internal validation was performed with bootstrap resampling, and independent hold-out validation was conducted with the independent hold-out testing set. Longitudinal follow-up of patients with chronic hepatitis B or cirrhosis was subsequently used to examine cumulative incidence across risk groups, thereby assessing the model’s ability to stratify future HBV-related HCC risk. A web-based calculator was developed to facilitate clinical application.

**Results:**

The study involved a cohort of 621 patients diagnosed with chronic HBV infection, divided into a training set of 373 (60.1%) patients and an independent hold-out testing set of 248 (39.9%) patients. Age (odds ratio [OR] 1.08, 95% CI 1.05‐1.12), ESPL1 expression (OR 1.01, 95% CI 1.00‐1.01), and log (alpha-fetoprotein) levels (OR 2.55, 95% CI 1.95‐3.33) were identified as independent predictors of HBV-related HCC. The model demonstrated excellent diagnostic discrimination, with a C-index of 0.922 in the training set and 0.958 in the independent hold-out testing set, coupled with strong calibration. Decision curve analysis revealed that the model consistently provided a higher net benefit across clinically relevant threshold probabilities. Subgroup analyses further validated the model’s high discriminative power, with C-index values ranging from 0.86 to 0.98, and no significant interactions were detected (all interaction *P* values > .10). Furthermore, the model demonstrated superior discriminatory power relative to 5 established HBV-related HCC risk scores, including REACH-B, GAG-HCC, CU-HCC, PAGE-B, mPAGE-B, and alpha-fetoprotein alone, with all pairwise comparisons yielding statistical significance (*P*<.001). For prognostic stratification, patients categorized as low risk, medium risk, and high risk had distinct 5-year cumulative HCC incidences of 5.1%, 21.1%, and 31.3%, respectively (*P*<.001).

**Conclusions:**

The ESPL1-based model may serve as both a diagnostic tool for differentiating patients with HCC from those with non-HCC and as a preliminary approach for risk stratification during follow-up. This dual role has the potential to support earlier detection and personalized monitoring. The web-based calculator improves accessibility and may facilitate future clinical integration.

## Introduction

### Global Burden and Clinical Challenges of Hepatitis B Virus–Related Hepatocellular Carcinoma

Hepatitis B virus (HBV), mainly spread through blood and bodily fluids, is a major global health concern and a leading cause of hepatocellular carcinoma (HCC), accounting for more than 50% of cases worldwide [1,2]. More than 200 million people have chronic HBV, of whom 15% to 25% at risk of developing liver cirrhosis or HCC. The prevalence and mortality of HBV-related HCC are projected to continue rising over the next two decades [3]. HCC increases public health challenges and places significant economic and emotional strain on patients and families [4]. Surgical resection is the best cure for early-stage HCC, but most patients are diagnosed at advanced stages because of the asymptomatic nature of early disease, rendering curative treatment infeasible. Thus, early diagnosis and risk stratification of high-risk patients are essential for improving HCC outcomes.

### Limitations of Current Risk Prediction Models

Current guidelines advocate for patients with chronic hepatitis B (CHB) to undergo a series of diagnostic tests every 3 to 6 months during antiviral therapy. However, challenges such as limited patient compliance, the low sensitivity of individual biomarkers, high costs, and inconsistent implementation across different health care settings impede the early detection of HCC [5-8]. A cost-effective approach to facilitate early HCC diagnosis and treatment involves the identification of high-risk individuals with chronic HBV infection and their regular monitoring. Risk prediction models provide personalized assessments of HCC risk for patients with CHB, thereby aiding physicians in improving patient adherence and optimizing screening and monitoring strategies [9,10]. Several models, including REACH-B, GAG-HCC, and PAGE-B, have been developed to assess HCC risk in patients with chronic HBV [11-16]. Nevertheless, these models primarily rely on conventional clinical parameters, limiting their generalizability, and they often fail to incorporate novel biomarkers or undergo validation for both diagnostic accuracy and longitudinal applicability.

### Objectives

Among the various potential biomarkers, the extra spindle pole bodies-like 1 (ESPL1) gene has garnered significant attention because of its involvement in genomic instability and tumor progression. Alterations in ESPL1, potentially induced by chronic viral infections or environmental carcinogens, may play a role in malignant transformation [17]. Our previous research identified frequent HBV S–ESPL1 fusion events in HBV-related HCC tissues and demonstrated that circulating ESPL1 protein levels can effectively differentiate early HCC from cirrhotic nodules [17,18]. Building upon these findings, this study pursued 2 primary objectives: first, to construct an ESPL1-based model capable of accurately distinguishing patients with HBV-related HCC from those with non-HCC, thereby demonstrating its diagnostic utility; and second, to substantiate its prognostic significance through longitudinal follow-up, stratifying patients with non-HCC according to their prospective risk of developing HCC. By integrating a biologically pertinent biomarker with real-world patient data, our model seeks to address the limitations of conventional methodologies and offer a clinically significant framework for both the early diagnosis and longitudinal risk stratification of HBV-related HCC.

## Methods

### Ethical Considerations

This study was approved by the Ethics Committee of the First Affiliated Hospital of Guangxi Medical University (2024-E614-01). The Ethics Committee of the First Affiliated Hospital of Guangxi Medical University waived written or oral informed consent of participants because there was no personally identifiable information in the article. The study followed the ethical standards outlined in the Declaration of Helsinki (World Medical Association Declaration of Helsinki 2013). All data were anonymized before analysis to protect patient privacy and confidentiality. No individual-level identifiers were included in the dataset, and all results are presented in aggregate form. No financial or other compensation was provided to participants in this study. Finally, no images or other materials that could potentially identify individual participants are presented in this manuscript or supplementary files.

### Study Design and Patients

This study was designed in 2 parts: (1) development of a diagnostic model to distinguish patients with HBV-related HCC from those with non-HCC (CHB and cirrhosis) and (2) longitudinal validation of its prognostic utility using follow-up data in patients with non-HCC.

This study is reported following the Transparent Reporting of a Multivariable Prediction Model for Individual Prognosis or Diagnosis reporting guideline checklist for developing and validating prediction models [19]. To minimize selection bias, all eligible patients with chronic HBV infection who were managed by our team from January 2012 to November 2023 were enrolled consecutively, adhering to stringent inclusion and exclusion criteria. Standardized follow-up protocols were implemented to accurately represent the natural clinical spectrum of HBV patients in our region. These protocols included assessments every 3 months during the first year of antiviral therapy, which comprised evaluations of HBV serological markers, HBV DNA levels, liver function tests, and alpha-fetoprotein (AFP) levels. Subsequently, follow-ups were conducted every 6 months, incorporating abdominal ultrasound. Patients diagnosed with cirrhosis or hepatic nodules were monitored every 3 months, with additional imaging modalities such as contrast-enhanced ultrasound, computed tomography, or magnetic resonance imaging recommended if nodules increased in size by more than 1.0 cm or if AFP levels rose.

To further reduce center-related bias, patients who met the inclusion criteria were divided based on their enrollment period into a training set and a temporally distinct testing set, rather than being allocated randomly. This approach provided an independent hold-out validation that more accurately simulates real-world application compared to random resampling. In addition, a subset of patients from the follow-up cohort, who had stored paired serum samples (1 at baseline and another after more than 5 years of follow-up), was included in a longitudinal analysis. These patients were managed under the same standardized protocol as the main cohort. Their paired samples were used to calculate predicted HCC risk values at both time points, thereby enabling an evaluation of the model’s capacity to capture dynamic changes in risk over time.

### Inclusion Criteria

The inclusion criteria have been described below.

#### Patients with Chronic HBV

These included individuals aged >18 years with CHB or HBV-related liver cirrhosis. CHB was defined as persistent HBsAg positivity for more than 6 months, detectable HBV DNA, and evidence of active liver disease, characterized by either persistently or intermittently elevated alanine aminotransferase (ALT) levels, histological findings of significant necroinflammation, or histological/noninvasive evidence of significant fibrosis (≥F2) [8]. HBV-related cirrhosis was diagnosed either histologically, based on liver biopsy consistent with cirrhotic changes, or clinically [8]. Clinical diagnosis required documented chronic HBV infection (current HBsAg positivity or HBsAg negativity with anti-HBc positivity and a history of HBsAg positivity >6 mo, with other causes excluded), together with at least two of the following 5 features: (1) imaging findings of cirrhosis and/or portal hypertension; (2) endoscopic evidence of esophageal or gastric varices; (3) liver stiffness measurement (LSM) consistent with cirrhosis, defined as ≥12.0 kPa when ALT <1×ULN or ≥17.0 kPa when ALT is 1‐5×ULN; (4) serum albumin <35 g/L or prothrombin time prolonged >3 seconds compared with controls; and (5) platelet count <100×10⁹/L.

#### Patients with HBV-related HCC

These included patients with pathologically confirmed HCC following partial hepatectomy. All HCC cases had chronic HBV infection as defined earlier, with no evidence of other etiologies such as hepatitis C, alcohol, or autoimmune liver disease.

#### Patients with Untreated HCC

These included patients with newly diagnosed HBV-related HCC who had not undergone prior curative or palliative therapies, including liver resection, transarterial chemoembolization, radiofrequency ablation, systemic therapy, or chemotherapy.

#### Adequate Frozen Serum Samples

There should have been the availability of at least 0.5 mL of stored serum per patient, collected before HCC treatment or during routine follow-up, and preserved at −40°C or lower in our serum biobank.

### Exclusion Criteria

The exclusion criteria were as follows: (1) patients co-infected with HBV and other infections such as hepatitis D, E, C, or HIV/AIDS; (2) patients with non-HBV-related cancers, including non-HBV-related HCC; (3) patients with liver disease from other causes, such as autoimmune liver disease; and (4) patients with decompensated liver cirrhosis or liver failure.

Peripheral venous blood samples (5 mL) were collected from patients in the CHB and cirrhosis of liver (LC) groups during outpatient visits and from the HBV-HCC group before surgical resection. Samples were centrifuged at 4000 rpm for 5 minutes, and the serum supernatant was stored at −40°C for subsequent analysis.

### Serum ESPL1 Levels Detection

Serum ESPL1 levels were measured using an Enzyme-Linked Immunosorbent Assay (ELISA) kit (Nova Lifetech Inc, Catalog No.: ELI-48263h) according to the manufacturer’s instructions. The procedure included standard and sample preparation, incubation, washing, addition of reagents, and optical density measurement. The absorbance (optical density value) of each sample was quantified using a microplate reader (BioRad, Co, Ltd, USA) at a wavelength of 450 nm. Three replicate wells were used for each sample, and the mean value was determined. The concentration of serum ESPL1 in the unknown samples was calculated based on the standard curve [20].

### Data Collection

Collected patient follow-up data included name, diagnosis, sex, family history of HCC or LC, age details (age at ESPL1 testing, antiviral treatment duration), antiviral drugs, HBV DNA levels, HBeAg status, liver nodules, and ALT, albumin (ALB), and AFP levels at ESPL1 testing.

A total of 16 variables were collected: 9 continuous (ESPL1, age at ESPL1 testing, antiviral treatment duration, HBV DNA, AFP, ALT, aspartate aminotransferase [AST], ALB, and liver stiffness) and 7 categorical (diagnosis, sex, family history of HCC/LC, treatment status, antiviral medication, HBeAg status, and liver nodules). To enhance data robustness and reduce outlier effects, a log transformation was applied to the HBV DNA and AFP data because of their wide range and extreme values.

### Missing Data

Missing data rates were 1.6% for AFP, 0.5% for ALB, and 18.7% for liver stiffness. Missingness was addressed using multiple imputation via the mice package in R (version 3.6.2, Institute for Statistics and Mathematics, Vienna, Austria) [21]. The missing data pattern was inspected, and imputations were generated under the assumption of missing at random using predictive mean matching, which preserves the observed data distribution. Five imputed datasets were created, pooled according to Rubin’s rules, and used for the primary analyses. To assess robustness, sensitivity analyses were conducted by comparing results from the pooled imputed dataset with those from the complete-case dataset, and each of the 5 individual imputed datasets. The retained predictors and their effect sizes were consistent across all analyses, indicating that missing data had minimal influence on the final model in Multimedia Appendix 1.

### Comparison With Established HCC Risk Models

To assess the comparative performance of the ESPL1-based Age-ESPL1-AFP (AEA) score, we conducted an evaluation against 5 established HBV-related HCC risk models (ie, REACH-B, GAG-HCC, CU-HCC, PAGE-B, and mPAGE-B [22]) using an independent hold-out testing cohort. These models were chosen due to the consistent availability of their requisite predictors within our dataset, facilitating direct implementation and ensuring a fair comparison. Each score was computed in accordance with its original published definition, with comprehensive details of the scoring algorithms provided in the Supplementary Materials in Multimedia Appendix 2. Subsequently, the predictive performance of these models was evaluated and juxtaposed with that of our model.

### Statistical Analysis

Statistical analysis was conducted using SPSS 26.0 and R 4.3.2 with rms and DynNom packages. Two-sided *P* values <0.05 were deemed significant. Normally distributed data are shown as “mean (SD)” and compared with *t* tests, whereas nonnormally distributed data are presented as [median (P25, P75)] and analyzed using the Wilcoxon rank-sum test. Categorical data are shown as n (%), and group differences were assessed with the *χ*^2^ test.

LASSO regression, a data mining technique, adds a penalty to traditional linear regression to simplify the model by reducing coefficient values, addressing multicollinearity, and preventing overfitting. It was used by the modeling group to initially select predictors for HBV-related HCC [23]. Variables with nonzero coefficients from the LASSO regression were refined using stepwise backward selection in multivariate logistic regression to determine the final predictors for HBV-related HCC risk. The primary objective was to establish a diagnostic tool that differentiates patients with HBV-related HCC from those with CHB/LC. Subsequently, to examine prognostic relevance, we applied the model to the non-HCC subgroup with longitudinal follow-up, assessing cumulative HCC incidence across risk strata. We then developed a clinical prediction model and an interactive web-based risk calculator using Shinyapps, designed exclusively for patients with chronic HBV infection or HBV-related cirrhosis, and not intended for use in the general population without HBV.

We assessed the model’s predictive performance by calculating discrimination and plotting calibration curves for both training and independent hold-out testing sets [24]. The C-index, ranging from 0.5 (no discrimination) to 1.0 (perfect prediction), was used to evaluate discrimination. Calibration was further evaluated using the Hosmer–Lemeshow goodness-of-fit test, which compares observed and predicted event rates across deciles of risk [25]. Calibration was also assessed using calibration curves and the Brier score, where lower scores indicate higher accuracy. We conducted 1000 bootstrap resampling iterations for internal validation of the model’s predictive performance. Optimal cutoff values for HBV-related HCC risk stratification were determined using X-tile software, a validated tool for biomarker assessment and outcome-based cutoff optimization [26]. These cutoffs were first applied to baseline predicted risk values in the longitudinal cohort, categorizing patients into low risk, medium risk, and high risk groups. The cumulative incidence of HCC within each risk group was then estimated using the Kaplan–Meier method, with differences between groups assessed by the log-rank test [27]. Clinical decision curves were used to evaluate the model’s clinical applicability. The main metric in the clinical decision curve was the net benefit, determined by the model’s true and false positive rates in predicting outcomes for the target population. The formula applied was as follows: net benefit=(true positives/N)−(false positives/N)×(Pt/(1−Pt)), with N as the total sample size and Pt as the model’s event probability threshold. By linking net benefits across various probabilities, we created a decision curve, where a higher net benefit signifies greater model value in clinical use [28].

## Results

### Patient Characteristics

**Figure 1.**
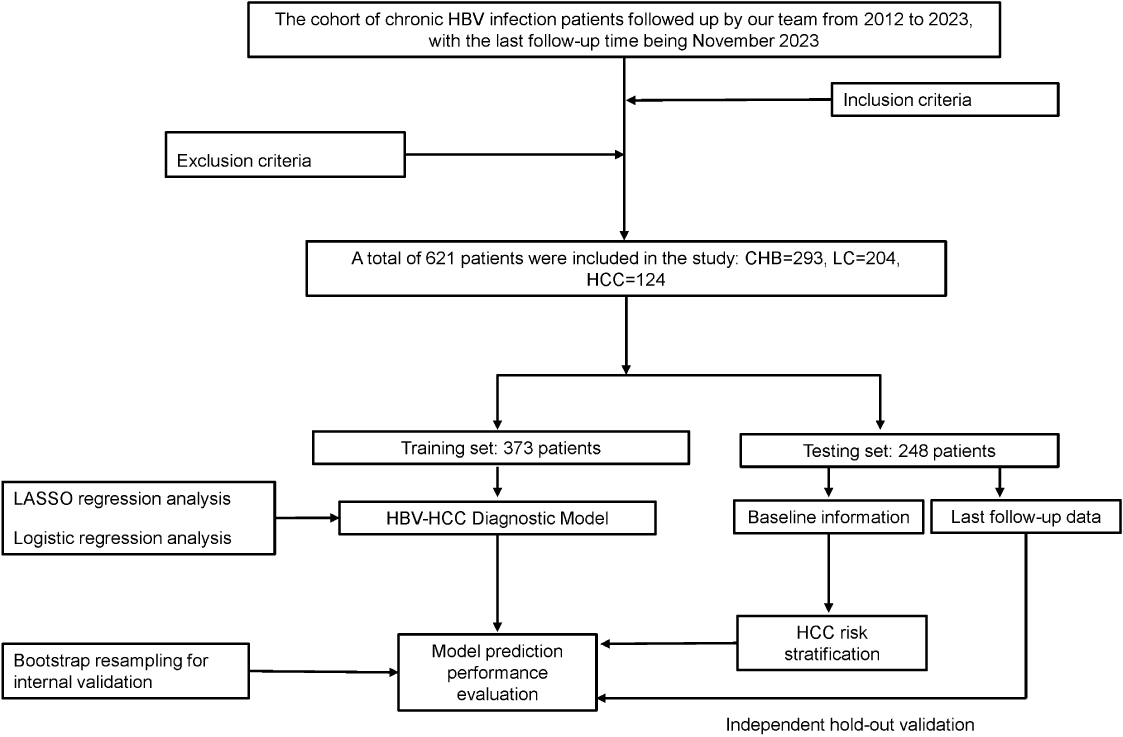
HBV-related HCC clinical prediction model research design flowchart. HBV: hepatitis B virus; HCC: hepatocellular carcinoma; CHB: chronic viral hepatitis B; LC: cirrhosis of liver.

A total of 621 patients with chronic HBV were included, comprising 373 in the training set (median follow-up: 2.6 years) and 248 in the independent hold-out testing set (median follow-up: 5.0 years), as shown in Figure 1. The overall median age was 46 years, with 467 males (75.2%) and 154 females (24.8%). At baseline, 293 patients (47.2%) had CHB, 204 (32.9%) had LC, and 124 (20.0%) had HCC. Clinical characteristics of the two cohorts are summarized in Table 1.

**Table 1.** Characteristics of patients included in the study.

Variable	Training set (n=371)	Independent hold-out testing set (n=248)
Sex, n (%)		
Male	277 (74.3)	190 (76.6)
Female	96( 25.7)	58 (23.4)
Age (y)	44 (37, 53)	48 (39, 54)
Family history of LC/HCC, n (%)		
No	180 (48.3)	162 (65.3)
Unknown	122 (32.7)	53 (21.4)
Yes	71 (19.0)	33 (13.3)
Previously treated, yes, n (%)	50 (13.4)	34 (13.7)
log (HBV DNA), median (P25, P75)	1 (1,3)	1 (1,1)
HBeAg positive, n (%)	118 (31.6)	57 (23.0)
AST^a^ (U/L), median (P25, P75)	31 (26, 41)	28 (25, 33)
ALT^b^ (U/L), median (P25, P75)	28 (20, 42)	23 (17, 30)
ALB^c^(g/L), median (P25, P75)	42.2 (40.1, 44.7)	42.3 (40.9, 44.4)
LSM^d^ (kPa), median (P25, P75)	6.6 (5.5, 11.5)	6.1 (5.2, 7.5)
ESPL1^e^ (ng/L), median (P25, P75)	270.7 (270.71, 350.06)	212.65 (153.94, 300.92)
AFP^f^ (ng/mL), median (P25, P75)	3.02 (2.06, 5.90)	2.50 (1.91, 3.59)
Antiviral treatment duration (months)	18 (0, 51)	104 (64, 141)
Antiviral drugs, n (%)		
Non–first-line nucleotides	53 (14.2)	26 (10.5)
First-line nucleotides^g^	62 (16.6)	145 (58.5)
Mixed medication	258 (69.2)	77 (31.0)
Liver nodule, n (%)		
Yes	203 (54.4)	169 (67.7)
No	170 (45.6)	79 (31.9)
Diagnosis, n (%)		
CHB^h^	157 (42.1)	135 (54.4)
LC^i^	126 (33.8)	75 (30.2)
HCC^j^	90 (24.1)	38 (15.3)

a AST: aspartate aminotransferase.

b ALT: alanine aminotransferase.

c ALB: albumin.

d LSM: liver stiffness measurement.

e ESPL1: extra spindle poles like 1.

f AFP: alpha-fetoprotein.

g First-line nucleotides: define entecavir or tenofovir as first-line nucleotide analog antiviral drugs.

h CHB: chronic hepatitis B viral.

i LC: liver cirrhosis.

j HCC: hepatocellular carcinoma.

During follow-up in the independent hold-out testing set, 38 patients (15.3%) developed HCC, with cumulative incidence rates of 2.4%, 9.3%, and 15.3% at 1, 3, and 5 years, respectively. Compared with the training set, patients in the independent hold-out testing set were older, had longer antiviral treatment duration, and exhibited higher ALT levels. They also showed lower HBV DNA, AFP, AST, and ESPL1 levels, fewer liver nodules, a lower prevalence of family history of HCC/LC, and different distributions of antiviral medication use.

### Construction of a Risk Prediction Model for HBV-Related HCC

Using LASSO regression, 9 predictors with non-zero coefficients were identified from an initial set of 16 variables, including gender, age, ESPL1, antiviral drugs, log (HBV DNA), Alb, AST, log (AFP), and liver nodules (Multimedia Appendix 3). Detailed information on these HCC-related predictors is provided in Table 2 (lambda.min=0.04091). Subsequently, a predictive model for predicting HBV-related HCC risk was developed using multivariable logistic regression, incorporating both the LASSO-selected variables and those identified through univariable logistic regression variables, as presented in Table 2. The final prediction model included age, ESPL1, and log (AFP) as independent predictors, and the regression coefficients are provided in Table 2. The estimated probability of HCC was calculated using the logistic regression equation: logit(P)=−8.9764+0.0802×age+0.0059×ESPL1+0.9365×log (AFP).

**Table 2.** Univariate and multivariate logistic regression analyses variables relating to HCC in the training set. Note: In multivariate logistic regression, variables such as first-line nucleotides and liver nodules produced extremely high odds ratios with confidence intervals of (0, Inf), consistent with quasi-complete separation. Under such conditions, maximum likelihood estimation becomes unstable, and the coefficients are not reliable; therefore, these variables were excluded from the final model.

Variable	Univariate analysis	*P* value	Multivariate analysis	*P* value
	OR (95% CI)		OR (95% CI)	
Sex				
Male	Reference		Reference	
Female	0.36 (0.19‐0.70)	.003	0.33 (0.08‐1.39)	.132
Age	3.40 (2.31‐5.01)	<.001	1.08 (1.05‐1.12)	<.001
ESPL1^a^	2.96 (2.17,4.03)	<.001	1.01 (1.00‐1.01)	<.001
Antiviral drugs				
Non–first-line nucleotides	Reference		Reference	
First-line nucleotides	73.17 (24.92‐214.82)	<.001	249,179 097.26 (0, Inf)	.990
Mixed medication	0.41 (0.14‐1.20)	.105	0.87 (0.13‐5.89)	.888
log (HBV^b^ DNA)	2.17 (0.71‐6.63)	.176	N/A	
ALB^c^	0.83 (0.62‐1.13)	.235	N/A	
AST^d^	1.04 (0.97‐1.11)	.248	N/A	
log (AFP^e^)	1.83 (1.57‐2.13)	<.001	2.55 (1.95‐3.33)	<.001
Liver nodule				
No	Reference		Reference	
Yes	221.95 (30.41‐1620.00)	<.001	3,944,282,432.72(0, Inf)	.988

a ESPL1: extra spindle poles like 1.

b HBV: hepatitis B virus.

c ALB: albumin.

d AST: aspartate aminotransferase.

e AFP: alpha-fetoprotein.

**Figure 2.**
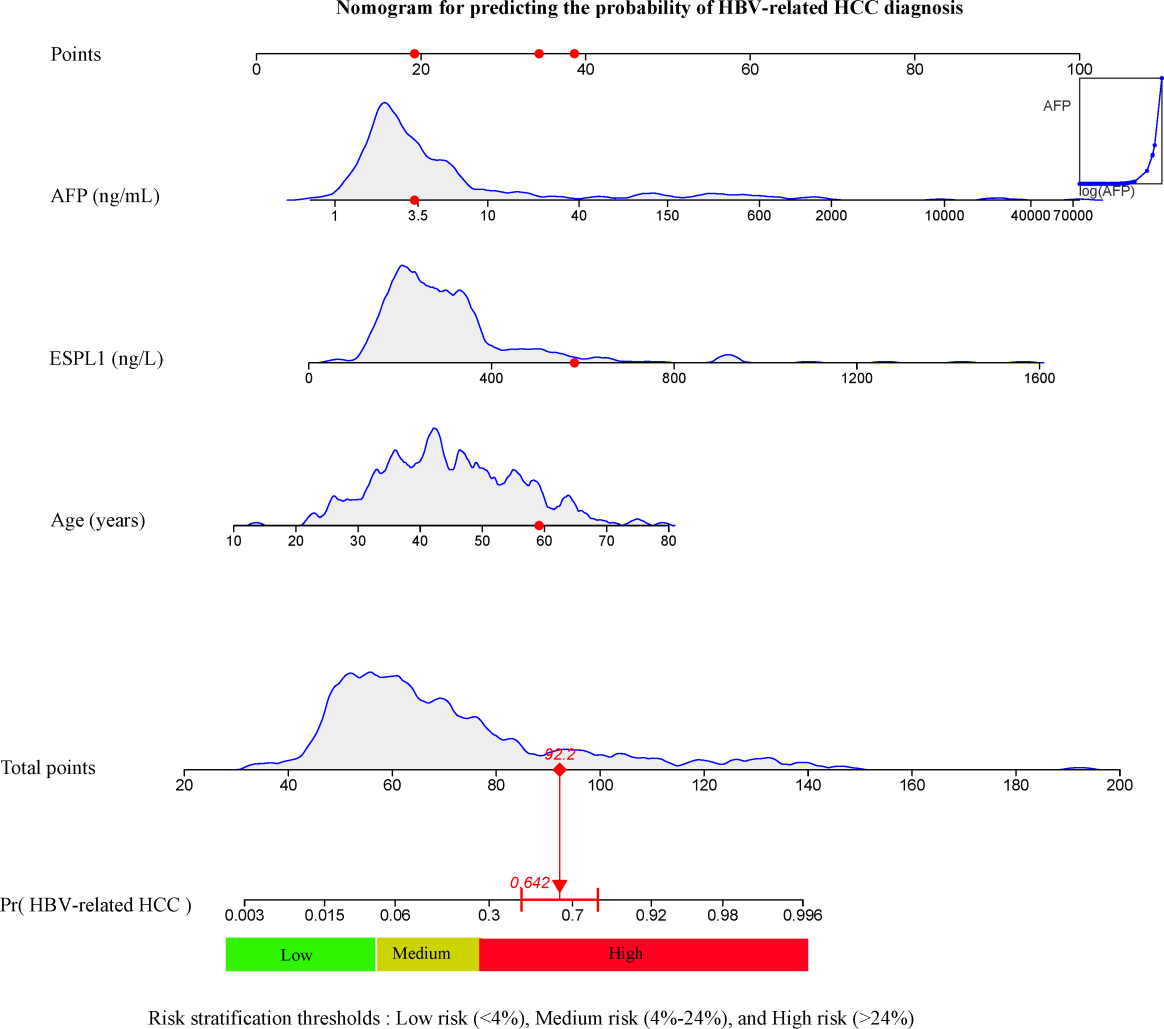
AEA score for predicting HBV-associated HCC risk was constructed based on the results of multivariate logistic regression analysis. Risk stratification thresholds were determined using X-tile: low risk (<4%), medium risk (4%‐24%), and high risk (>24%), as illustrated by the colored bar at the bottom of the figure. This is an example of calculating the probability of HBV-related HCC risk in patients. The irregular curves corresponding to each variable in the figure represent the distribution of variable values in the modeling group. On the basis of the corresponding variable, a score can be obtained, and the scores are added to get a total score. Finally, the total score can be used to calculate the corresponding HCC risk probability. In this illustrative case of a patient with pathologically confirmed HBV-related HCC, the red dots for AFP, ESPL1, and age indicate the patient’s respective values: AFP=3.32 ng/mL, ESPL1=581.60 ng/L, and age=59 years. The corresponding scores (19+39+34.2) yield a total of 92.2, which translates into a predicted HCC risk probability of 64.2%. AFP: alpha-fetoprotein; ESPL1: extra spindle poles like 1; HBV: hepatitis B virus; HCC: hepatocellular carcinoma.

**Figure 3.**
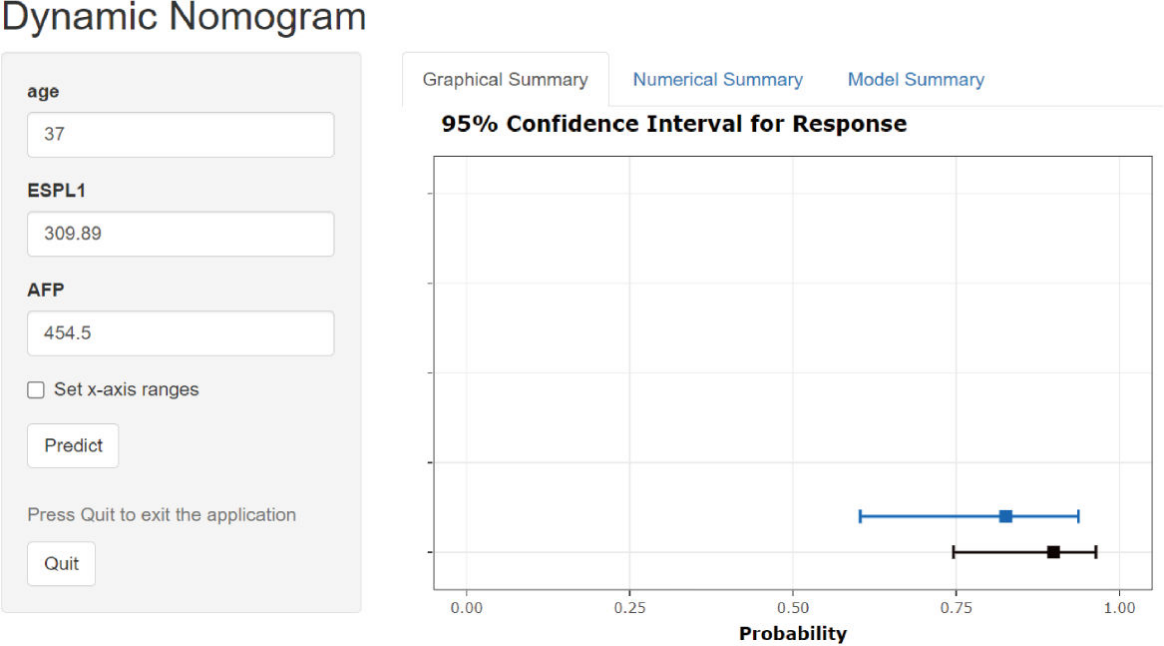
Example of HBV-related HCC risk probability web calculator. Age is in years, AFP in ng/mL, and ESPL1 in ng/L. AFP: alpha-fetoprotein; ESPL1: extra spindle poles like 1; HBV: hepatitis B virus; HCC: hepatocellular carcinoma.

A risk model for predicting HBV-related HCC was developed using age, ESPL1, and AFP (AEA score) from multivariable logistic regression analysis, as depicted in Figure 2. Each variable has a score, and their sum estimates HCC risk. For example, in a 59-year-old male patient with pathologically confirmed HBV-related HCC who had an AFP level of 3.32 ng/mL and an ESPL1 level of 581.60 ng/L, the calculated total score corresponded to a predicted HCC risk probability of 64.2%. Conversely, in a 45-year-old male patient with chronic HBV infection and imaging evidence of cirrhotic nodules but no HCC diagnosis, with an ESPL1 level of 252.50 ng/L and an AFP level of 3.09 ng/mL, the calculated score indicated a predicted HCC risk probability of 7.47%. These contrasting examples illustrate how the AEA score can distinguish between patients at markedly different levels of HCC risk within the HBV population. For easier clinical use, we developed a web calculator [29]. In this, we can access the URL, input the necessary data, and click “Predict” to view the patient’s current HCC risk, as shown in Figure 3.

Sensitivity analyses, which compared the pooled imputed dataset, the 5 individual imputed datasets, and the complete-case dataset, yielded highly consistent results. The same independent predictors were retained, and similar effect sizes were observed across all methodologies, as detailed in Multimedia Appendix 1.

### Evaluation of HBV-Related HCC Diagnostic Model Performance

#### Internal Validation

**Figure 4.**
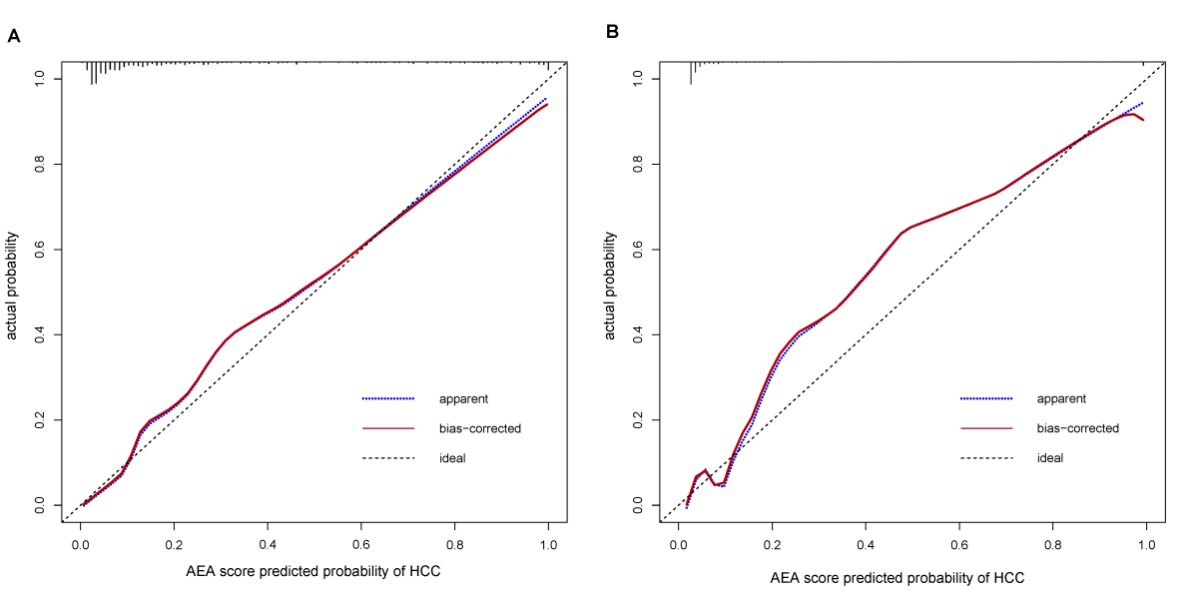
Calibration curves of the AEA score in the training set (A) and independent hold-out testing set (B). The dashed line indicates perfect calibration, the dotted line represents the apparent performance, and the solid line shows the bias-corrected performance after 1000 bootstrap resamples. Short vertical ticks at the top of each panel denote the distribution of patients according to predicted HCC risk (rug plot). HCC: hepatocellular carcinoma.

In the training set, the Hosmer–Lemeshow test yielded a *P* value of .596, suggesting an absence of evidence for model overfitting. The model demonstrated a C-index of 0.922 (95% CI 0.890–0.954), with an internally validated C-index of 0.923 (95% CI 0.890–0.950). It exhibited a consistency of 0.83, sensitivity of 0.89, specificity of 0.82, a positive predictive value of 0.606, a negative predictive value of 0.959, and a Youden Index of 0.71, indicating excellent discriminatory power. Furthermore, the model surpassed the performance of AFP alone (C-index 0.605) and ESPL1 alone (C-index 0.773) in predicting HCC risk. The calibration curve (Figure 4A) demonstrated a strong concordance between predicted and observed HCC incidence, with a slope of 0.93, a mean squared error of 0.023, and a Brier score of 0.08. Collectively, these findings suggest that the model provides both precise and dependable risk estimation, which is crucial for early clinical decision-making.

To further evaluate robustness, subgroup analyses were conducted across clinically pertinent categories, including sex, antiviral drug usage, treatment history, family history of LC/HCC, and HBeAg status in Multimedia Appendix 4. The model consistently exhibited high discriminatory performance, with C-index values ranging from 0.86 to 0.98, and no significant interactions were detected (all *P* values for interaction >.10). Owing to the absence of HCC events among noncirrhotic patients during the follow-up period, subgroup analysis stratified by cirrhosis status was not feasible. Collectively, these findings affirm that the ESPL1-based model maintains stable predictive accuracy across diverse patient subgroups, underscoring its reliability for broad clinical application.

#### Independent Hold-Out Validation

In the independent hold-out testing set, the model demonstrated robust performance, evidenced by a C-index of 0.958 (95% CI 0.929–0.988) and an adjusted C-index of 0.958 (95% CI 0.926–0.986). Additionally, the model exhibited a consistency of 0.92, a sensitivity of 0.87, a specificity of 0.94, a positive predictive value of 0.696, a negative predictive value of 0.976, and a Youden Index of 0.81. The calibration curve (Figure 4B) confirmed consistency between predicted and observed outcomes, with a slope of 0.91, mean squared error of 0.027, and a Brier score of 0.05. These findings support the robustness and clinical applicability of the model, suggesting that its predictions remain reliable when applied to independent patient data.

### Comparison of AEA Score With Other HBV-Related HCC Risk Scores

The AEA score consistently exhibited superior discriminatory power compared to the 5 established HBV-related HCC risk models (REACH-B, GAG-HCC, CU-HCC, PAGE-B, and mPAGE-B). Within our cohort, the C-index of the AEA score was significantly greater than that of each comparator model, with all pairwise comparisons achieving statistical significance (*P*<.001, Multimedia Appendix 5). In addition, the discriminative ability of AFP alone was limited (C-index=0.61, 95% CI 0.56‐0.65), further highlighting the incremental value of incorporating ESPL1 into the AEA score. These results underscore the enhanced predictive value of integrating ESPL1 into risk assessment, surpassing traditional demographic and clinical variables.

### Validation of AEA Score for HBV-Related HCC in a Longitudinal Cohort

**Figure 5.**
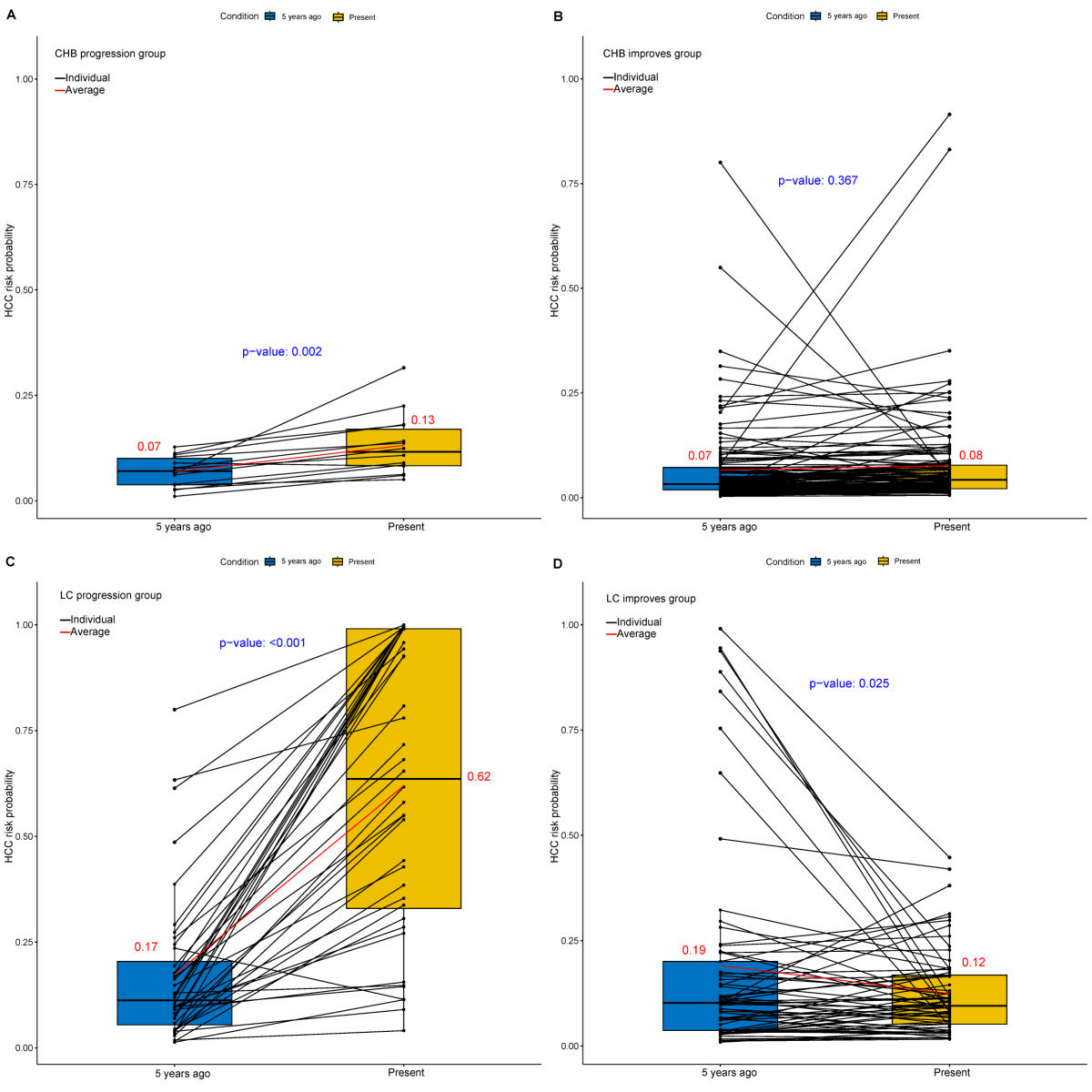
Trend chart of AEA score predicting HCC risk changes with the condition of patients with chronic HBV infection. HBV: hepatitis B virus; HCC: hepatocellular carcinoma.

The longitudinal analysis confirmed that the ESPL1-based AEA score dynamically reflected disease trajectories: predicted HCC risk remained stable or significantly decreased in patients with clinical improvement, whereas it rose markedly in those with disease progression. Patients with CHB who exhibited clinical improvement (n=129) maintained stable risk values over a 3-year period (0.07 vs 0.08, *P*=0.367). In contrast, those who experienced disease progression (n=14) showed a significant increase in risk values (0.07 vs 0.13, *P*=.002; Figure 5A–B). Similarly, among patients with cirrhosis, those demonstrating clinical improvement (n=65) experienced a significant reduction in predicted risk (0.19 vs 0.12, *P*=.025), whereas patients who developed HCC during follow-up (n=40) exhibited a substantial increase in risk probability, rising from 0.17 to 0.62 (*P*<.001; Figure 5C–D). These results underscore the model’s capability not only to estimate baseline risk but also to detect significant changes corresponding with clinical outcomes, thereby affirming its utility for dynamic risk monitoring.

**Figure 6.**
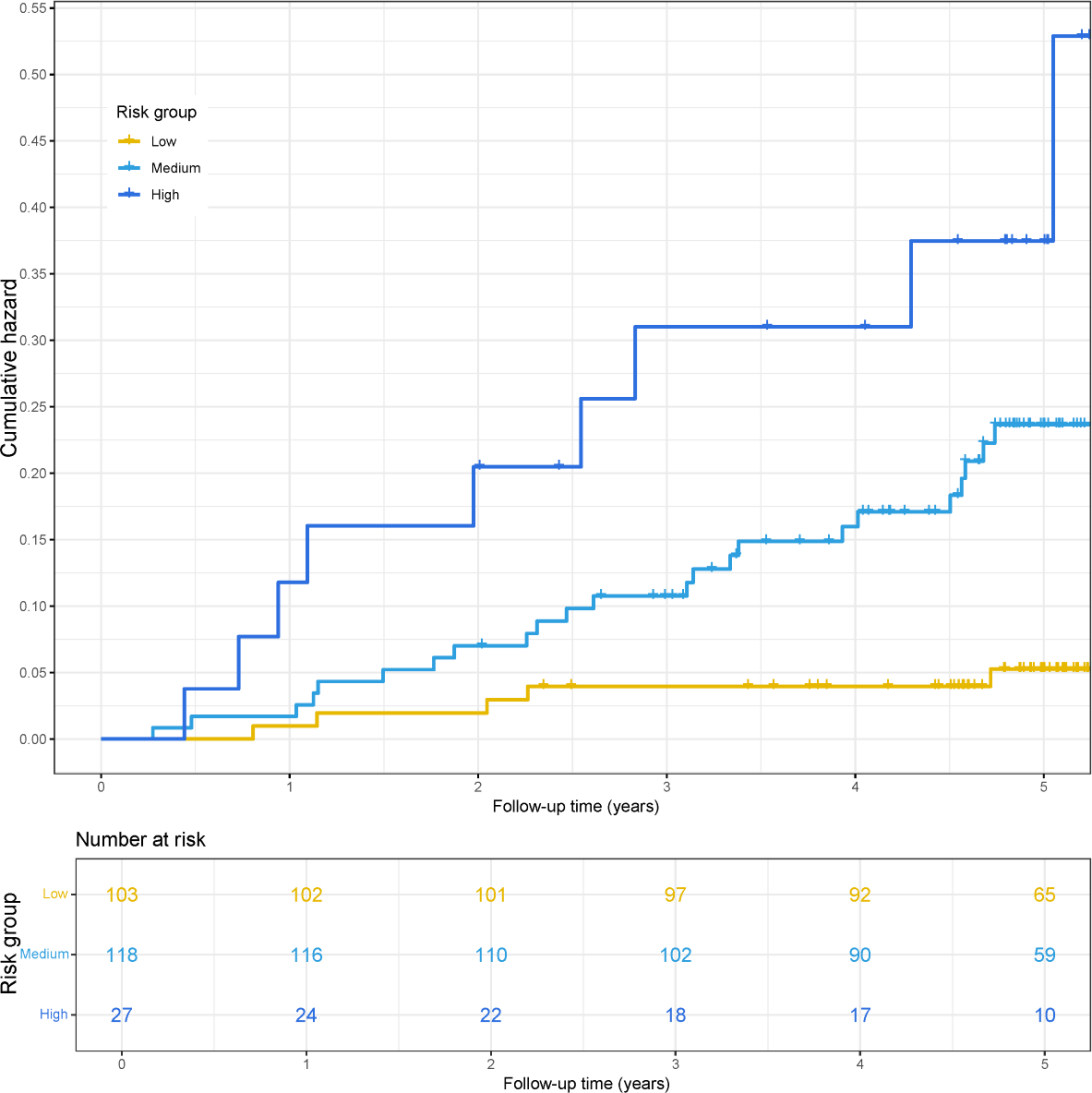
Cumulative incidence curve of HBV-related HCC in longitudinal cohort. HBV: hepatitis B virus; HCC: hepatocellular carcinoma.

Using the X-tile tool, 3 optimal cutoff points (4% and 24%) were identified for stratifying HBV-related HCC risk. Application of these thresholds in the independent hold-out validation cohort (n=248) categorized patients into low risk (45.6%), medium risk (47.5%), and high risk (10.9%) groups (Figure 6). Kaplan–Meier analysis revealed significant differences in cumulative HCC incidence among groups, with 5-year rates of 5.1% in the low-risk group, 21.1% in the medium-risk group, and 31.3% in the high-risk group (*P*<.001). This separation highlights the ability of the model to statistically distinguish relative risk levels among patients, which may support risk-adapted surveillance strategies in future studies.

As shown in Multimedia Appendix 6, the distribution of clinical outcomes at the end of follow-up varied markedly across the 3 risk strata in the independent hold-out testing set. In the high-risk group, most patients had progressed to HBV-related HCC by the end point (88%), whereas the medium-risk group comprised a mixture of CHB, LC, and HCC cases. In contrast, the low-risk group at follow-up was composed predominantly of patients with CHB, and no HCC events were observed in this category. This clear gradient in outcome distribution demonstrates the model’s ability to stratify patients according to their likelihood of HBV-related HCC development and underscores its potential to inform surveillance strategies.

### The Clinical Application Value of HBV-Related HCC Prediction Model

**Figure 7.**
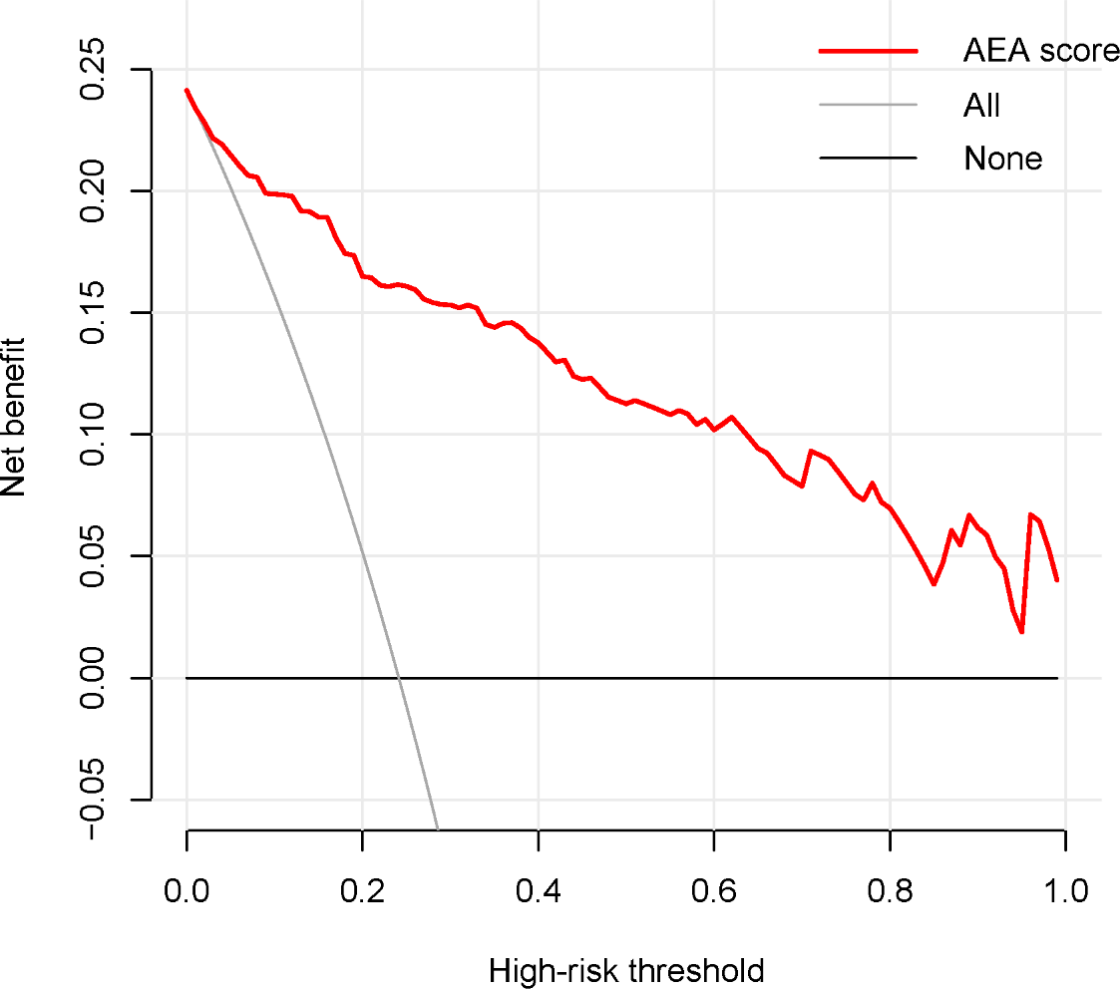
Clinical decision curve of the AEA score in HCC diagnosis. Net benefit is the primary outcome observed in the clinical decision curve. It is calculated based on the true positive rate and false positive rate of the model’s prediction for the outcome in the target population. The formula is as follows: net benefit=(true positives/N)–(false positives/N)×(Pt/(1−Pt)), where N is the total sample size and Pt is the probability threshold used by the model to distinguish between the occurrence and nonoccurrence of the event. Connecting the net benefit values at different probability thresholds forms the decision curve. A higher net benefit indicates greater practical value of the model in real-world applications.

As shown in Figure 7, decision curve analysis indicated that our AEA score consistently provided greater net benefit than either the “All” and “None” strategies when the risk threshold is above 0.01. This finding implies that the model has the potential to enhance surveillance efficiency by improving case detection across clinically relevant thresholds. Nevertheless, all risk groups continue to face nonnegligible risks, necessitating ongoing monitoring in alignment with current guidelines.

## Discussion

### Principal Findings

Chronic HBV infection can lead to liver inflammation and fibrosis, which may progress to HCC. Early detection of HCC significantly enhances prognosis; however, the asymptomatic nature of its early stages poses challenges for timely diagnosis. Recent technological advancements [30] and the identification of novel biomarkers, in conjunction with clinical features, are expected to enhance the accuracy and feasibility of early HCC detection. Building on our previous research, we developed an AEA score that integrates the newly identified ESPL1 protein biomarker with long-term clinical data from patients with chronic HBV infection. The AEA score demonstrated superior performance in both accuracy and calibration compared to existing HCC risk models. In addition, subgroup analyses indicated that the ESPL1-based model maintained high discriminatory accuracy across various factors, including sex, antiviral regimen, treatment history, family history of LC or HCC, and HBeAg status, thereby affirming its robustness across diverse clinical contexts.

### Comparison to Prior Work

In this study, the ESPL1-based AEA score demonstrated consistently superior predictive accuracy compared with 5 widely used HBV-related HCC risk models (ie, REACH-B, GAG-HCC, CU-HCC, PAGE-B, and mPAGE-B). As our study was conducted in Guangxi, a region with a particularly high incidence of HBV-related HCC, these findings emphasize that conventional models may not fully reflect risk profiles in this population. By integrating ESPL1, the AEA score provides additional discriminatory value beyond demographic and clinical variables, enabling more accurate risk stratification in high-burden settings. With respect to individual biomarkers, AFP and PIVKA-II remain widely used in clinical practice but have well-recognized limitations in sensitivity and specificity for early HCC detection. In our comparative analysis within the same cohort, ESPL1 demonstrated significantly stronger discriminatory ability than both AFP and PIVKA-II (C-index: 0.923 vs 0.572 and 0.788, respectively), underscoring its potential to distinguish early HCC from cirrhotic nodules [17,18,31]. Although LSM reflects fibrosis and cirrhosis, it was not retained as an independent predictor in our multivariate analysis, suggesting limited incremental value when combined with ESPL1 and clinical factors in this population. Taken together, these findings highlight that while conventional models and biomarkers provide useful prognostic information, they remain insufficient for precise early HCC risk prediction in patients with chronic HBV, especially in high-incidence regions such as Guangxi. By incorporating ESPL1 with readily available clinical variables, the AEA score delivers both superior discrimination and appears to provide incremental value for risk stratification in populations with a high burden of HBV-related HCC. Nevertheless, these observations should be interpreted cautiously, as our study was based on a single-center cohort and requires external confirmation.

Our study used a cross-sectional design combined with LASSO regression to identify key predictors, thereby reducing redundancy and confounding relative to traditional Cox survival models. This methodology facilitated the selection of the most informative variables and the development of a parsimonious logistic regression model, ultimately enhancing both predictive accuracy and stability. The final model, which included age, serum ESPL1 levels, and log (AFP), exhibited strong discrimination and calibration in both the training and testing sets. In contrast, other variables commonly cited as predictors of HCC in previous studies, such as serum albumin [13,32,33], HBV DNA [11,12], ALT [11], and LSM [34], did not maintain independent significance in our multivariate analysis. A possible explanation for this finding is that early-stage HCC induces only subtle changes in biochemical markers, whereas the widespread use of antiviral therapy diminishes the predictive value of HBV DNA levels. Similarly, although LSM reflects fibrosis and cirrhosis, it may not offer additional discriminatory power once ESPL1 and AFP are accounted for.

### Biological Rationale

Our prior study revealed that the expression of the HBV S–ESPL1 fusion gene persists despite long-term antiviral therapy, underscoring its specific role in hepatocarcinogenesis [17,35]. Mechanistically, ESPL1 encodes separase, a cysteine protease that plays a critical role in chromatid segregation during anaphase. Dysregulation of ESPL1 results in chromosomal instability, a well-established driver of malignant transformation. Under conditions of chronic viral infection and prolonged carcinogenic stress, integration of HBV into the ESPL1 locus can produce HBV S–ESPL1 fusion transcripts, potentially enhancing oncogenic signaling and compromising genomic integrity. Previous studies have demonstrated that mutations, overexpression, or viral fusion events involving ESPL1 contribute to uncontrolled cell proliferation, defective DNA repair, and the activation of tumor-promoting pathways, including p53 inhibition and Wnt/β-catenin signaling [36-38]. Beyond HCC, overexpression of ESPL1 has also been implicated in breast, prostate, osteosarcoma, and endometrial cancers, highlighting its role as a broadly relevant oncogenic driver [39-41]. From a clinical perspective, these biological mechanisms elucidate the enhanced efficacy of ESPL1 in comparison to traditional biomarkers. AFP, the most commonly used biomarker, demonstrates limited sensitivity in the early stages of HCC, frequently remaining below diagnostic thresholds and exhibiting a stronger correlation with tumor burden rather than the molecular initiation of malignancy [18,42]. Conversely, serum levels of ESPL1 protein may indicate early oncogenic events induced by HBV integration and genomic instability, thereby capturing risk signals before the overt formation of tumors. In our study, AFP alone did not reach conventional diagnostic thresholds; however, the log (AFP) maintained independent predictive significance. Notably, when combined with patient age and serum ESPL1 levels, the resultant AEA score demonstrated excellent discrimination (C-index 0.92) and was robustly validated in external cohorts. Collectively, these findings suggest that ESPL1 enhances predictive accuracy in a statistically significant manner and serves as a mechanistically substantiated biomarker that encapsulates the molecular mechanisms underlying HBV-induced hepatocarcinogenesis. This dual benefit—biological plausibility combined with robust clinical efficacy—advocates for its incorporation into risk prediction models to facilitate earlier and more precise identification of patients with high-risk HBV.

### Clinical Implications

Cirrhotic nodules, a family history of liver cirrhosis or HCC, and the use of non–first-line antiviral agents are well-recognized clinical risk factors for HCC development, and their identification remains important for patient management. In our univariate analysis, all 3 factors were significantly associated with HCC risk. However, these associations lost statistical significance in the multivariate model. This phenomenon likely reflects that their predictive influence is relatively modest once stronger predictors, such as age, serum ESPL1, and AFP, are accounted for and may also be partly explained by correlations among variables (eg, cirrhotic nodules reflecting fibrosis already captured by ESPL1 or AFP). Our subgroup analyses further confirmed that the ESPL1-based model maintained robust discrimination across strata of sex, antiviral treatment regimen, prior treatment status, family history of LC/HCC, and HBeAg status, with no significant interactions detected. These findings suggest that the diminished significance of these factors in the multivariate model does not reflect instability, but rather their limited incremental predictive value beyond the key predictors. Nevertheless, given their established clinical relevance, these factors should not be disregarded in practice, and future studies with larger multicenter cohorts and nonlinear modeling approaches may help clarify their contribution to HCC risk prediction.

Accurate HCC risk prediction in patients with chronic HBV is essential during follow-up. Tumor heterogeneity and complex serum biomarkers pose challenges. Unlike models using AFP or PIVKA-II, our HBV-related HCC risk model based on ESPL1 integrates patient disease status and serological marker characteristics. This enables personalized HCC risk assessments to inform patient follow-up strategies. Our study shows that patients with an HCC risk probability over 24% are at high risk. This was validated externally, where patients with chronic HBV who developed HCC had an average risk of 62%, much higher than the 17% baseline. This confirms our model’s effectiveness in predicting HCC occurrence. The model’s risk values allow patients to be categorized into low risk, medium risk, and high risk groups, with significant differences in 3-year and 5-year HCC incidence rates. These findings suggest that ESPL1 may improve early identification of patients at elevated risk; however, their implications for guiding follow-up strategies need to be validated in larger multicenter cohorts. In the internet age, online calculators enable quick risk assessments. Such tools may facilitate predicting HCC risk exclusively in patients with chronic HBV infection or HBV-related cirrhosis, supporting individualized follow-up strategies. However, their role in routine clinical decision-making remains exploratory and requires further validation.

Clinical prediction models are crucial for assessing HCC risk in patients with chronic HBV, aiding physicians in predicting risk and guiding treatment. However, traditional evaluation metrics such as discrimination and calibration often fall short in reflecting clinical benefits at varying risk thresholds [43-45]. Discrimination metrics such as AUC assess a model’s predictive accuracy but do not confirm its clinical usefulness. To validate the clinical utility of the HBV-related HCC risk prediction model based on ESPL1, we used clinical decision curve analysis instead of multicenter, large-sample prospective studies, which are time-consuming and involve many uncertainties. Clinical decision curves reveal potential clinical benefits at various risk thresholds and calculate net benefits [28]. An ideal curve should show positive net benefits across clinically relevant thresholds. Our analysis indicates that when the threshold probability is over 1%, the model achieves a positive net benefit. In addition, the 4% and 24% thresholds used to categorize patients into low risk, medium risk, and high risk groups in our HBV-RELATED HCC model are within this range. These results provide insight into possible clinical relevance but cannot substitute for large-scale, multicenter prospective validation. Our findings suggest that the model may be clinically informative, yet confirmation in diverse populations is warranted before widespread adoption.

In addition to its predictive accuracy, the practical feasibility of ESPL1 testing is a critical consideration for clinical translation. Serum ESPL1 levels can be measured through a standard ELISA assay, which is cost-effective and comparable in price to AFP testing. Although this suggests potential feasibility, reproducibility across laboratories and consistency in different health care systems have not yet been established. Therefore, widespread clinical implementation should await further multicenter validation and assay standardization. Furthermore, we developed a web-based calculator that can be accessed on computers and mobile devices, allowing clinicians to easily input routinely available variables, including ESPL1, to obtain individualized risk estimates. At this stage, the calculator should be regarded as a prototype tool to illustrate the model’s potential application rather than a ready-to-use clinical instrument.

### Limitations

This study has several limitations. First, all patients were recruited from a single tertiary hospital in Guangxi, which may introduce selection bias and limit representativeness compared with broader, community-based populations. Second, as the study population was restricted to a high-incidence HBV region, the global generalizability of ESPL1 as a biomarker remains uncertain. Third, ESPL1 levels were quantified using a single ELISA kit under standardized laboratory conditions, and interlaboratory variability may affect reproducibility. Fourth, although major demographic, clinical, and virological factors were included, residual confounding from unmeasured variables (eg, lifestyle behaviors, alcohol consumption, aflatoxin exposure, or other environmental risks) cannot be excluded. We attempted to mitigate these limitations using strict inclusion criteria, internal and independent hold-out validation, and robust statistical modeling; however, multicenter prospective studies are needed to confirm these findings.

### Future Directions

Future research should focus on multicenter, geographically diverse cohorts to validate the ESPL1-based model, standardize assays across laboratories, and evaluate integration into clinical practice. In addition, combining ESPL1 with other emerging biomarkers or imaging modalities may further enhance predictive accuracy. Although the model demonstrated prognostic value during longitudinal validation, it was primarily developed for diagnostic purposes. Therefore, larger multicenter prospective cohorts will be needed to confirm its long-term predictive performance and ensure generalizability. Further work should also aim to standardize ESPL1 assays, assess cost-effectiveness in different health care systems, and evaluate patient and clinician acceptance. Finally, mechanistic studies on ESPL1 in HBV-related hepatocarcinogenesis may reveal therapeutic targets and extend its utility beyond risk stratification.

### Conclusions

The ESPL1-based AEA score exhibits enhanced predictive accuracy relative to existing HBV-related HCC risk models, indicating significant potential for individualized risk stratification. By incorporating both biomarker and clinical features, this model could facilitate earlier detection of HCC and enable personalized surveillance strategies. However, larger multicenter prospective studies across diverse populations are necessary to validate its broader applicability.

## Supplementary material

10.2196/78354 Multimedia Appendix 1 Sensitivity analysis of predictors for HBV-related HCC risk using multiple imputation and complete-case datasets. HBV: hepatitis B virus; HCC: hepatocellular carcinoma.

10.2196/78354 Multimedia Appendix 2 Established HCC risk models. HCC: hepatocellular carcinoma.

10.2196/78354 Multimedia Appendix 3 LASSO regression analysis selected predictors. (A) The tuning parameter (λ) for the LASSO model was selected using 10-fold cross-validation based on the minimum criterion. Dashed lines represent the optimal values using λ.min and 1− λ.SE. (B) LASSO regression analysis was conducted on 19 features, generating a coefficient plot using the log (λ min) sequence through 10-fold cross-validation. The optimal λ (λ.min = 0.04091) identified 16 predictors with nonzero coefficients.

10.2196/78354 Multimedia Appendix 4 Subgroup analysis of the ESPL1-based model in the training set. Forest plot showing the discriminatory performance (C-index and 95% CI) of the ESPL1-based model across clinically relevant subgroups, including sex, antiviral drugs, treatment history, family history of LC/HCC, and HBeAg status. As no HCC events occurred among patients with noncirrhosis during follow-up, subgroup analysis stratified by cirrhosis status could not be performed. ESPL1: extra spindle poles like 1; HCC: hepatocellular carcinoma; LC: cirrhosis of liver.

10.2196/78354 Multimedia Appendix 5 Comparison of C-index of the AEA score with other existing risk scores in the training set.

10.2196/78354 Multimedia Appendix 6 Distribution of patients in different HBV-related HCC risk layers in the external testing set. HBV: hepatitis B virus; HCC: hepatocellular carcinoma.

## Abbreviations

AFP: alpha-fetoprotein
AFP-L3: alpha-fetoprotein variant-L3
ALB: albumin
ALT: alanine aminotransferase
AST: aspartate aminotransferase
AUC: area under the ROC curve
CHB: chronic viral hepatitis B
ESPL1: extra spindle poles like 1
HBV: hepatitis B virus
HCC: hepatocellular carcinoma
LC: cirrhosis of liver
LSM: liver stiffness measurement
NA: nucleotide
PIVKA-Ⅱ: protein-induced by vitamin K absence/antagonist-Ⅱ
PLT: platelet
PT: prothrombin time
TBil: total bilirubin
