# Supplementary material for: Development and Validation of an Extra Spindle Pole Bodies–like 1–Based Diagnostic and Prognostic Model for Hepatitis B Virus–Related Hepatocellular Carcinoma: Retrospective Cohort Study
Source: JMIR Med Inform. 2025 Oct 22;13:e78354. doi: 10.2196/78354 (PMC12543211; doi:10.2196/78354)
Supplement: Multimedia Appendix 1 [file medinform-v13-e78354-s001.docx]

| Table S1 Sensitivity analysis of predictors for HBV-related HCC risk using multiple imputation and complete-case datasets | | | |
| --- | --- | --- | --- |
| Model Metric | C-index (95% CI) | | |
|  | Age | ESPL1 | Log (AFP) |
| Interpolation data 1 OR | 1.06 (1.04–1.09) | 1.01 (1.00–1.01) | 2.70 (2.21–3.38) |
| Interpolation data 1 *P* value | <.001 | <.001 | <.001 |
| Interpolation data 2 OR | 1.06 (1.04–1.09) | 1.01 (1.00–1.01) | 2.63 (2.16–3.27) |
| Interpolation data 2 *P* value | <.001 | <.001 | <.001 |
| Interpolation data 3 OR | 1.06 (1.04–1.09) | 1.01 (1.00–1.01) | 2.51 (2.07–3.10) |
| Interpolation data 3 *P* value | <.001 | <.001 | <.001 |
| Interpolation data 4 OR | 1.06 (1.04–1.09) | 1.01 (1.00–1.01) | 2.65 (2.17–3.31) |
| Interpolation data 4 *P* value | <.001 | <.001 | <.001 |
| Interpolation data 5 OR | 1.06 (1.04–1.09) | 1.01 (1.00–1.01) | 2.47 (2.05–3.04) |
| Interpolation data 5 *P* value | <.001 | <.001 | <.001 |
| complete-case dataset OR | 1.06 (1.04–1.09) | 1.01 (1.00–1.01) | 2.59 (2.08–3.24) |
| complete-case dataset *P* value | <.001 | <.001 | <.001 |
| Interpolation summary data OR | 1.08 (1.05–1.12) | 1.01 (1.00–1.01) | 2.55 (1.99–3.40) |
| Interpolation summary data *P* value | <.001 | <.001 | <.001 |
| Note. Multivariate logistic regression was performed to identify independent predictors of HBV-related HCC. Missing data were handled using multiple imputation with predictive mean matching (PMM) in the mice package (m = 5 imputations). Results are presented for each imputed dataset (Interpolation data 1–5), the complete-case dataset excluding patients with missing values (Complete-case dataset), and the pooled summary estimates across imputations (Interpolation summary data). The pooled dataset represents the final analysis dataset used for model development. Odds ratios (ORs) and 95% confidence intervals (CIs) are shown. Consistent results across imputed and complete-case datasets confirm the stability of the model and indicate that missing data had minimal impact on the conclusions. | | | |
