# Supplementary material for: Development and Validation of an Extra Spindle Pole Bodies–like 1–Based Diagnostic and Prognostic Model for Hepatitis B Virus–Related Hepatocellular Carcinoma: Retrospective Cohort Study
Source: JMIR Med Inform. 2025 Oct 22;13:e78354. doi: 10.2196/78354 (PMC12543211; doi:10.2196/78354)
Supplement: Multimedia Appendix 2 [file medinform-v13-e78354-s002.docx]

| Table S2-1. REACH-B model | | | |
| --- | --- | --- | --- |
| **Predictors** | **Category** | **Coefficient** | **Score** |
| **Sex** | Female | 0 | 0 |
|  | Male | 0.78798 | 2 |
| **Age** | Per 1 year | 0.09859 |  |
|  | 30-34 |  | 0 |
|  | 35-39 |  | 1 |
|  | 40-44 |  | 2 |
|  | 45-49 |  | 3 |
|  | 50-54 |  | 4 |
|  | 55-60 |  | 5 |
|  | 60-65 |  | 6 |
| **ALT(U/L)** | <15 | 0 | 0 |
|  | 15-44 | 0.38823 | 1 |
|  | ≥45 | 0.96311 | 2 |
| **HBeAg** | Negative | 0 | 0 |
|  | Positive | 0.81308 | 2 |
| **HBVDNA** | <300(undetectable) | 0 | 0 |
| (copies/ml) | 300-9999 | 0.11648 | 0 |
|  | 10000-99999 | 1.31467 | 3 |
|  | 100000-999999 | 2.27028 | 5 |
|  | ≥10^6^ | 2.09258 | 4 |

| **Table S2-2. GAG-HCC model** | | |
| --- | --- | --- |
| **Predictors** | **Category** | **Score** |
| **Sex** | Female | 0 |
|  | Male | 14 |
| **Age** | Per 1 year | Age |
| **HBVDNA(Copies/ml in log)** | Per 1 log | 3*log |
| **Cirrhosis** | Presence | 33 |
|  | Absence | 0 |

**Formula:**

GAG-HCC score=14*sex(male=1, female=0)+age(in years)+3*HBVDNA levels(Copies/ml in log)+33*cirrhosis(presence=1, absence=0).

| **Table S2-3. CU-HCC model** | | |
| --- | --- | --- |
| **Predictors** | **Category** | **Score** |
| **Age** | >50 | 3 |
|  | ≤50 | 0 |
| **ALB(g/L)** | ≤35 | 20 |
|  | >35 | 0 |
| **Tbil(μmol/l)** | >18 | 1.5 |
|  | ≤18 | 0 |
| **Cirrhosis** | Yes | 15 |
|  | No | 0 |
| **HBVDNA(Log Copies/ml)** | ≤4 | 0 |
|  | 4-6log | 1 |
|  | >6 | 4 |

| **Table S2-4A. PAGE-B model** | | | |
| --- | --- | --- | --- |
| **Predictors** | **Category** | **Coefficient** | **Score** |
| **Sex** | Female | 0 | 0 |
|  | Male | 1.4901 | 6 |
| **Age** | Per 1 year | 0.0527 |  |
|  | 16-29 |  | 0 |
|  | 30-39 |  | 2 |
|  | 40-49 |  | 4 |
|  | 50-59 |  | 6 |
|  | 60-69 |  | 8 |
|  | ≥70 |  | 10 |
| **PLT** | ≥200 | 0 | 0 |
|  | 100-199 | 1.5744 | 6 |
|  | <100 | 2.3258 | 9 |

|  | |
| --- | --- |
| **Table S2-4B. PAGE-B risk score table** | |
| **Score** | **5 years risk (%)** |
| 0 | 0.09 |
| 2 | 0.15 |
| 4 | 0.25 |
| 6 | 0.43 |
| 8 | 0.73 |
| 9 | 0.94 |
| 10 | 1.23 |
| 12 | 2.07 |
| 13 | 2.68 |
| 14 | 3.48 |
| 15 | 4.5 |
| 16 | 5.82 |
| 17 | 7.5 |
| 18 | 9.64 |
| 19 | 12.37 |
| 20 | 15.79 |
| 21 | 20.04 |
| 22 | 25.25 |
| 23 | 31.53 |
| 25 | 47.36 |

| **Table S2-5A. mPAGE-B model** | | |
| --- | --- | --- |
| **Predictors** | **Category** | **Score** |
| **Sex** | Female | 0 |
|  | Male | 2 |
| **Age** | <30 | 0 |
|  | 30-39 | 3 |
|  | 40-49 | 5 |
|  | 50-59 | 7 |
|  | 60-69 | 9 |
|  | ≥70 | 11 |
| **PLT** | ≥250 | 0 |
|  | 200-250 | 2 |
|  | 150-200 | 3 |
|  | 100-150 | 4 |
|  | <100 | 5 |
| **ALB(g/dl)** | ≥4 | 0 |
|  | 3.5-4 | 1 |
|  | 3-3.5 | 2 |
|  | <3 | 3 |

|  | | | | | |
| --- | --- | --- | --- | --- | --- |
| **Table S2-5B. mPAGE-B model risk score table** | | | | | |
| **Score** | **3 years risk (%)** | **5 years risk (%)** | **Score** | **3 years risk (%)** | **5 years risk (%)** |
| 0 | 0.14 | 0.23 | 11 | 7.76 | 12.8 |
| 1 | 0.2 | 0.33 | 12 | 11.06 | 18.01 |
| 2 | 0.29 | 0.48 | 13 | 15.62 | 25.01 |
| 3 | 0.41 | 0.7 | 14 | 21.83 | 34.12 |
| 4 | 0.6 | 1.01 | 15 | 30.03 | 45.39 |
| 5 | 0.87 | 1.46 | 16 | 40.41 | 58.4 |
| 6 | 1.25 | 2.12 | 17 | 52.78 | 71.96 |
| 7 | 1.81 | 3.05 | 18 | 66.31 | 84.17 |
| 8 | 2.62 | 4.4 | 19 | 79.34 | 93.09 |
| 9 | 3.77 | 6.31 | 20 | 89.84 | 97.92 |
| 10 | 5.42 | 9.01 | 21 | 96.37 | 99.64 |
