# Supplementary figures and images for: Development and Validation of an Extra Spindle Pole Bodies–like 1–Based Diagnostic and Prognostic Model for Hepatitis B Virus–Related Hepatocellular Carcinoma: Retrospective Cohort Study
Source: JMIR Med Inform. 2025 Oct 22;13:e78354. doi: 10.2196/78354 (PMC12543211; doi:10.2196/78354)

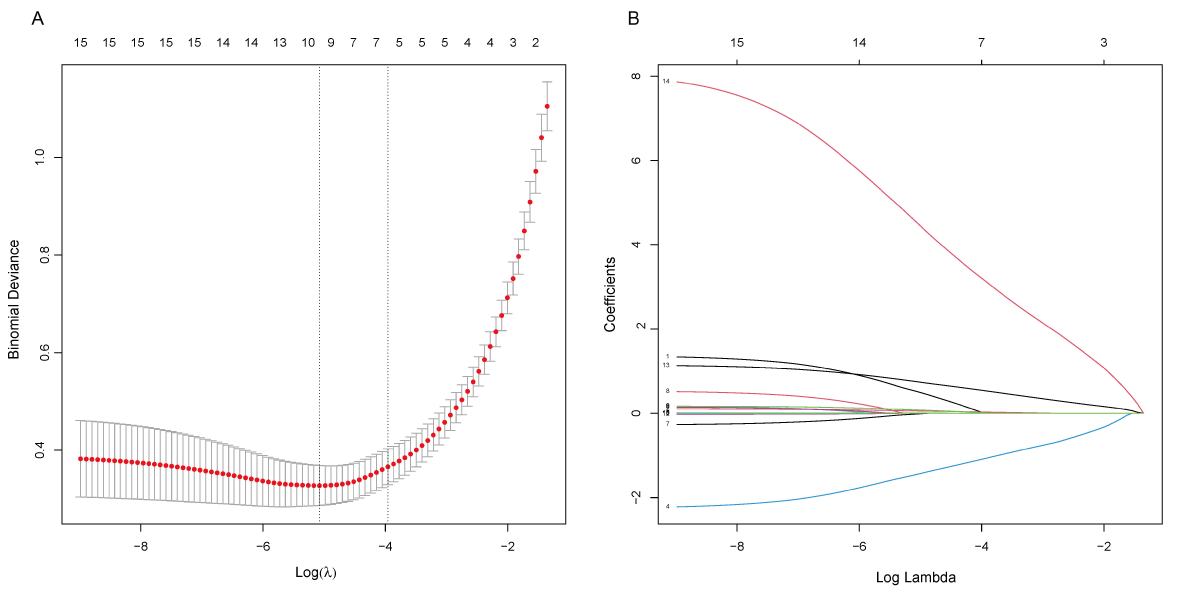

Supplement: Multimedia Appendix 3 [file medinform-v13-e78354-s003.png]

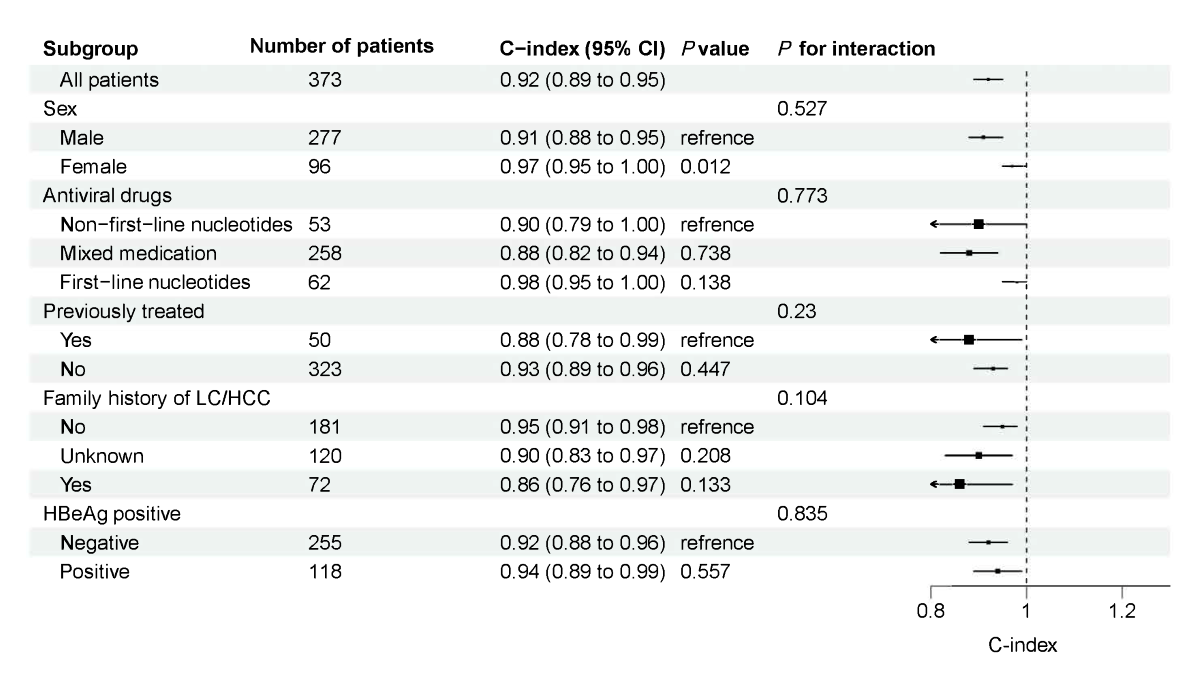

Supplement: Multimedia Appendix 4 [file medinform-v13-e78354-s004.png]
