# Supplementary material for: Development and Validation of an Extra Spindle Pole Bodies–like 1–Based Diagnostic and Prognostic Model for Hepatitis B Virus–Related Hepatocellular Carcinoma: Retrospective Cohort Study
Source: JMIR Med Inform. 2025 Oct 22;13:e78354. doi: 10.2196/78354 (PMC12543211; doi:10.2196/78354)
Supplement: Multimedia Appendix 5 [file medinform-v13-e78354-s005.docx]

| Table S3. Comparison of C-index of the AEA score with other existing risk scores in the training set | |
| --- | --- |
| Risk scores | C-index (95% CI) |
| AEA score | 0.95 (0.93–0.99) |
| REACH-B | 0.63（0.54,0.72） |
| GAG-HCC | 0.71（0.64,0.77） |
| CUHCC | 0.84（0.79,0.89） |
| PAGE-B | 0.71（0.65,0.77） |
| mPAGE-B | 0.77（0.71,0.83） |
| The C-index of the AEA score was significantly higher than those of the other HCC risk scores [p<0.001 (vs. REACH-B), p <0.001 (vs. GAG-HCC); p<0.001 (vs. CUHCC); p<0.001 (vs. PAGE-B); and p<0.001 (vs. mPAGE-B)] | |
