# Supplementary material for: Development and Validation of an Extra Spindle Pole Bodies–like 1–Based Diagnostic and Prognostic Model for Hepatitis B Virus–Related Hepatocellular Carcinoma: Retrospective Cohort Study
Source: JMIR Med Inform. 2025 Oct 22;13:e78354. doi: 10.2196/78354 (PMC12543211; doi:10.2196/78354)
Supplement: Multimedia Appendix 6 [file medinform-v13-e78354-s006.docx]

| Table 4S Distribution of patients in different HBV-related HCC risk layers in the external testing set | | | |
| --- | --- | --- | --- |
| Risk Stratification | CHB（n=135） | LC（n=75） | HCC（n=38） |
| Low risk, n (%) | 64（47.4） | 12（16.0） | 0（0） |
| Medium risk, n (%) | 64（47.4） | 54（72.0） | 5（13.2） |
| High risk, n (%) | 7（5.2） | 9（12.0） | 33（88.8） |
